# Chronic Central Leptin Infusion Promotes an Anti-Inflammatory Cytokine Profile Related to the Activation of Insulin Signaling in the Gastrocnemius of Male Rats

**DOI:** 10.3390/biomedicines10071465

**Published:** 2022-06-21

**Authors:** Vicente Barrios, Santiago Guerra-Cantera, Álvaro Martín-Rivada, Sandra Canelles, Ana Campillo-Calatayud, Eduardo Arilla-Ferreiro, Laura M. Frago, Julie A. Chowen, Jesús Argente

**Affiliations:** 1 Department of Endocrinology, Hospital Infantil Universitario Niño Jesús, Instituto de Investigación La Princesa, E-28009 Madrid, Spain; santiago.guerra@estudiante.uam.es (S.G.-C.); amrivada@salud.madrid.org (Á.M.-R.); sandra.canelles@salud.madrid.org (S.C.); acampilloc@salud.madrid.org (A.C.-C.); laura.frago@uam.es (L.M.F.); julieann.chowen@salud.madrid.org (J.A.C.); 2 Network Center for Biomedical Research on Obesity and Nutrition, Instituto de Salud Carlos III (CIBEROBN, ISCIII), E-28009 Madrid, Spain; 3 Department of Pediatrics, Faculty of Medicine, Universidad Autónoma de Madrid, E-28029 Madrid, Spain; 4 Biochemical Unit, Department of Biological Systems, Faculty of Medicine, Universidad de Alcalá, E-28871 Alcalá de Henares, Spain; eduardo.arilla@uah.es; 5 IMDEA Food Institute, CEI UAM + CSIC, E-28049 Madrid, Spain

**Keywords:** adiponectin, cytokine, gastrocnemius, inflammation, leptin, NEFA, signaling

## Abstract

Leptin is involved in the modulation of insulin signaling in peripheral tissues, being closely associated with changes in lipid metabolism. This adipokine modifies inflammatory pathways that can interact with insulin targets in peripheral organs; however, the mechanisms remain unclear. Inflammatory and insulin signaling targets, cytokines, adiponectin, irisin and non-esterified fatty acid (NEFA) levels and enzymes of fatty acid anabolism were studied in the gastrocnemius of chronic centrally infused leptin (L), pair-fed and control rats. The phosphorylation of signal transducer and activator of transcription 3 (STAT3) and c-Jun *N*-terminal kinase (JNK) was reduced in L rats (59% and 58%, respectively). The phosphorylation of the insulin receptor and Akt and adiponectin and irisin content was increased in L rats (154%, 157%, 308% and 329%, respectively). The levels of glucose-6-phosphate dehydrogenase, the mRNA content of acetyl Co-A carboxylase and NEFA concentrations were diminished in the muscles of L rats (59%, 50% and 61%, respectively). The activation of JNK correlated positively with STAT3 phosphorylation, tumoral necrosis factor-α and NEFA and negatively with irisin and Akt phosphorylation. These data suggest that the activation of insulin signaling targets and a decrease in NEFA content are associated with a reduction in muscle inflammation parameters, suggesting that leptin may integrate these pathways.

## 1. Introduction

Skeletal muscles make up the largest tissue in non-obese people, are key tissues for glucose disposal and can also act as a fuel reservoir in some circumstances, such as during fasting [1]. Muscle is the main target tissue for insulin in glucose metabolism, while in obesity, it can use non-esterified fatty acids (NEFAs) as an energy substrate. This situation diminishes the rates of glucose consumption and also inhibits the insulin-stimulated glucose uptake provoked by the deposition of fatty acid metabolites in muscle cells [2].

The adipokine leptin acts centrally to regulate insulin signaling and raises skeletal muscle insulin sensitivity by increasing the activity of insulin signaling targets, including insulin receptor substrates (IRSs). Insulin stimulation leads to the activation of phosphatidylinositol (PI) 3-kinase (PI3K), producing PI-triphosphate, which triggers Akt activation and glucose uptake [3]. After the PI-triphosphate-mediated translocation of Akt to the plasma membrane, Akt is phosphorylated on Ser473 and Thr308, with both phosphorylations being needed for the highest activation of this kinase [4].

The relationship between the signaling and actions of leptin and insulin is more evident in obesity, where skeletal muscle shows resistance to leptin actions, contributing to the accumulation of lipids including intramuscular long-chain fatty acyl-CoA [5]. The dysregulation of fatty acid metabolism and the augmentation in NEFAs in muscle is clearly connected to the development of insulin resistance in this tissue [6], whereas pharmacological [7] and lifestyle interventions can enhance peripheral insulin sensitivity accompanied by a reduction in muscle lipids associated with an improvement in the fatty acid oxidation rate [8].

The development of insulin resistance is linked to an augmentation in proinflammatory cytokines [9]. Long-chain fatty acids generate endoplasmic reticulum stress which promotes inflammation. The expression of cytokines, such as interleukin (IL)2 and IL-6 and tumoral necrosis factor (TNF)α, among others, activates c-jun *N*-terminal kinase (JNK). This kinase can phosphorylate IRS1 on serine residues to trigger insulin resistance [10]. Additionally, the phosphorylation of signal transducer and activator of transcription 3 (STAT3) can increase p38 mitogen-activated protein kinase (p38-MAPK), and in turn activates JNK [11].

Conversely, some adipokines and myokines, such as adiponectin and irisin, increase the insulin-sensitizing effects in skeletal muscle, such as glucose uptake and fatty acid oxidation [12,13]. Irisin suppresses inflammation and promotes macrophage polarization from type M1 to M2 and raises Akt phosphorylation [14], while decreased irisin concentrations are related to insulin resistance in high-fat-diet-exposed mice [15].

We hypothesized that increased leptin concentrations could improve insulin signaling in gastrocnemius and that this is related to modifications in inflammation and lipid levels. Most reports of leptin’s effects on insulin sensitivity have been performed in the soleus [16,17,18]. Therefore, the aims of this study were to compare the effect of chronic central leptin infusion with pair-fed rats on the probable changes in the activation of inflammatory targets and tissue cytokine profile and their relationship with modifications in insulin signaling and lipid metabolism in the gastrocnemius of Wistar male rats. Lastly, the potential contribution of this tissue to the peripheral cytokine pattern and insulin-sensitizing molecules was also evaluated. Pair-fed rats were included to distinguish between the effects of leptin and those only due to a decrease in food intake. 

## 2. Materials and Methods

### 2.1. Materials

All reagents were purchased from Merck (Darmstadt, Germany) unless otherwise mentioned. Specific antibodies against actin were from Thermo Fisher Scientific (Waltham, MA, USA), protein tyrosine phosphatase 1B (PTP1B) from Merck and suppressor of cytokine signaling 3 (SOCS3) from Proteintech Europe (Manchester, UK). The Immun-Star Western C kit (ECL) was from Bio-Rad Laboratories (Hercules, CA, USA). The corresponding secondary antibodies conjugated with horseradish-peroxidase, the high-capacity cDNA kit and the TaqMan gene expression assay were purchased from Thermo Fisher Scientific (Waltham, MA, USA).

### 2.2. Animals

All procedures were carried out according to the local ethics committee and complied with Royal Decree 53/2013 relating to the protection of experimental animals and with the European Communities Council Directive (2010/63/EU). This project was approved by the Ethical Committee of Animal Experimentation of the Universidad de Alcalá (PROEX018/16, 14 June 2016). The number of animals used in this study was reduced to the minimum needed. Male Wistar rats (250 ± 10 g) acquired from Harlan Laboratories (Barcelona, Spain) were housed separately under controlled dark–light cycles (12 h/12 h), temperature (22 °C) and humidity (50%) and had access to water ad libitum. Animals were anesthetized by employing 4 mg of ketamine/100 g body weight (bw) and 0.5 mg of xylazine/100 g bw throughout the surgical procedures [19,20].

### 2.3. Experimental Design

Fifteen rats were anesthetized, positioned in a stereotaxic apparatus and treated as previously reported [21,22] after a fasting period of 12 h. Concisely, a cannula, coupled to an osmotic minipump (Alzet, Durect Corp., Cupertino, CA, USA) which contained either vehicle (saline 0.9% plus 1% serum albumin) or leptin, was implanted into the right cerebral ventricle. The leptin was previously dissolved in saline plus 1% bovine serum albumin (BSA), and the rats were treated icv for 14 days with either vehicle or leptin (12 μg/day). In addition, as this leptin treatment decreases food intake, we included a pair-fed group that received the same quantity of food ingested by the leptin-treated group the day before. This resulted in the following groups: vehicle icv (control, C), pair-fed rats with vehicle icv (PF) and leptin icv (L) with five animals per group. After 14 days of treat-ment, the rats were sacrificed via decapitation at 8.00 h after a 12 h fasting period, and the gastrocnemius was subsequently dissected and processed. Trunk blood was centrifuged at 1800× *g* for 10 min at 4 °C, and the serum was stored at −70 °C until it was processed.

### 2.4. Serum and Tissue Non-Esterified Fatty Acid (NEFA) Levels

Concentrations of NEFA were determined by using a colorimetric kit (Wako Chemicals, Neuss, Germany), following the enclosed instructions. For the determination of their levels in muscle, an extraction of total lipids was performed following the method of Folch et al. [23]. The average coefficients of variation were lower than 10%.

### 2.5. Tissue Homogenization and Protein Quantification

For the immunodetection of adiponectin, pThr308Akt, Akt, fractalkine, interferon γ (IFN-γ), interleukin (IL)-2, IL-4, IL-6, IL-10, phosphorylated (p) insulin receptor (p-IR), pSer636-IRS1, pTyr-IRS1, IRS1, irisin, pThr183/Tyr185-JNK, JNK, pThr180/Tyr182-p38 MAPK, p38MAPK, pSer2448 mammalian target of rapamycin (pSer2448-mTOR), mTOR, pSer536-nuclear factor kappa B (pSer536-NFkB), NFkB, pTyr705 signal transducer and activator of transcription 3 (pTyr705STAT3), pSer727STAT3, STAT3 and tumor necrosis factor α (TNF-α), 50 mg of gastrocnemius was homogenized on ice in 400 µL of lysis buffer (Merck). The lysates were frozen 12 h at −70 °C and subsequently centrifuged at 12,000× *g* for 5 min at 4 °C. Supernatants were stored at −70 °C until they were assayed. Protein levels were determined using the Bradford method (Bio-Rad Laboratories).

### 2.6. ELISAs

#### 2.6.1. Adiponectin

The levels of adiponectin in serum and muscle were determined by using an ELISA kit from Merck. Adiponectin in the sample or standard was captured using a monoclonal antibody in the microtiter plate, and after subsequent washing, a second biotinylated anti-adiponectin antibody was added. After washing, an HRP-streptavidin conjugated to a biotinylated antibody was added. Finally, tetramethylbenzidine (TMB) was added, and the absorbance at 450 nm was measured.

#### 2.6.2. Irisin

Irisin was measured in serum and tissue lysates using an ELISA kit from BioVendor (Brno, Czech Republic). After the binding of irisin to a polyclonal antibody in the microplate and washing, an HRP conjugate was added. After incubation and washing, TMB was added, and the absorbance was quantified.

#### 2.6.3. Phosphorylation of Insulin Receptor

The ELISA from Assay Solution (Woburn, MA, USA) detects phosphorylated insulin receptor β protein. After incubation with lysates of gastrocnemius, the ligand was bound by the monoclonal antibody in the microplate. Following washing, a detection antibody linked to biotin was added. A streptavidin-HRP complex was added and after washing, TMB was used, and the absorbance at 450 nm was measured. Intra- and inter-assay variation coefficients were lower than 10% in all assays.

### 2.7. Western Blotting

Western blotting was performed as previously described [24]. In total, 20 µg of protein was resolved on 10% sodium-dodecyl-sulphate-denaturing polyacrylamide gels, transferred to polyvinylidene difluoride membranes, then incubated with PTP1B or SOCS3 antibodies. Peroxidase activity was detected by using an ECL system (Bio-Rad Laboratories), and the chemiluminescent signal was calculated with ImageQuant Las 4000 Software (GE Healthcare Life Sciences, Barcelona, Spain). Gel-loading variabilities were normalized with actin.

### 2.8. Multiplexed Bead Immunoassays

The phosphorylated and total protein levels of Akt, IRS1, JNK, mTOR and NFkB, as well as the concentrations of fractalkine, IFN-γ, IL-2, IL-4, IL-6, IL-10 and TNF-α in gastrocnemius were measured using multiplexed bead immunoassays (Bio-Rad Laboratories and Merck) following the manufacturer’s recommendations. Beads conjugated to antibodies and muscle lysates (50 μL each) were incubated, and antibody conjugated to biotin was added. Afterwards, beads were incubated with streptavidin–phycoerythrin. At least 50 beads per variable were examined in the Bio-Plex suspension array system 200 (Bio-Rad Laboratories). Raw data (median fluorescence intensity, MFI) were evaluated with the Bio-Plex Manager Software 4.1 (Bio-Rad Laboratories). The mean intra- and inter-assay coefficients of variation were 8.3% and 11.9%, respectively.

### 2.9. Enzyme Activity Assays

#### 2.9.1. Glucose-6-Phosphate Dehydrogenase (G6PD)

The activity of this dehydrogenase (EC 1.1.1.49) was assayed with a kit from Sigma-Aldrich. After the homogenization of the gastrocnemius in phosphate-buffered saline and centrifugation, supernatants were incubated at 37 °C with master reaction mix, and absorbance at 450 nm was measured. 

#### 2.9.2. Malic Enzyme

The activity of this enzyme (EC 1.1.1.40) was determined using the method of Geer et al. [25]. Diluted supernatants were incubated at 25 °C with triethanolamine buffer, malic acid and nicotinamide adenine dinucleotide phosphate (NADP), and absorbance was examined at 340 nm.

### 2.10. RNA Purification and Real-Time PCR Analysis

Total RNA was extracted from the gastrocnemius according to the Tri-Reagent protocol [26]. The reverse transcription reaction was carried out on 2 μg of RNA using a high-capacity cDNA archive kit (Applied Biosystems, Foster City, CA, USA). Real-time PCR was performed in an ABI Prism 7000 Sequence Detection System (Applied Biosystems) using TaqMan PCR Master Mix. PCRs were performed in a total volume of 50 μL with 25 μL of the reverse transcription reagents. A TaqMan gene expression assay was employed for acetyl-CoA carboxylase (ACC)β (Rn00588290_m1). Relative gene expression comparison was carried out using an invariant endogenous control (actin, Rn00667869_m1). The ΔΔCT method was used for relative quantification.

### 2.11. Statistical Analysis

The analysis of all data was carried out using one-way ANOVA followed by Bonferroni’s post hoc tests using Statview (Statview 5.01, SAS Institute, Cary, NC, USA) software. Data are represented as mean ± standard error of the mean (SEM). Linear regression analysis was employed to assess the relationships between specific parameters. Values were considered significantly different when the p value was less than 0.05. Graphs were prepared using GraphPad Prism 8 (San Diego, CA, USA) software.

## 3. Results

### 3.1. General Characteristics of the Experimental Groups

We previously confirmed that food intake and body weight gain were decreased in the PF and L groups. Additionally, we also reported that serum leptin was increased in rats treated centrally with leptin [24]. The weight of gastrocnemius was higher (*p* < 0.05) in the L group compared to PF rats (1.09 ± 0.07, 0.99 ± 0.06 and 1.33 ± 0.07, data expressed as percentage of total body weight in C, PF and L groups, respectively), and muscle NEFA levels did not change in PF rats and were diminished (*p* < 0.01) in L rats (16.0 ± 0.6, 18.2 ± 1.9 and 10.7 ± 0.5 mg/dL in C, PF and L groups, respectively).

### 3.2. Serum Levels of Cytokines

**Figure 1:**
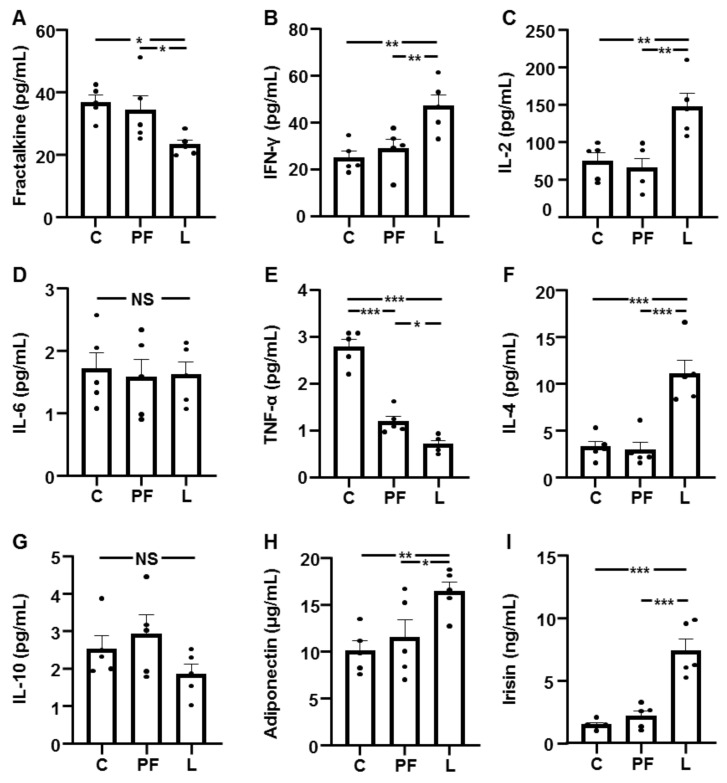
Circulating concentrations of cytokines. Serum fractalkine (**A**), interferon (IFN)γ (**B**), interleukin (IL)2 (**C**), IL-6 (**D**), tumor necrosis factor (TNF)α (**E**), IL-4 (**F**), IL-10 (**G**), adiponectin (**H**) and irisin (**I**) levels in control rats (C), pair-fed rats (PF) and rats receiving chronic intracerebroventricular leptin infusion (L). Data are presented as means ± SEM. NS, non-significant. * *p* < 0.05, ** *p* < 0.01, *** *p* < 0.001; one-way ANOVA followed by Bonferroni’s test. Dots represent the individual data.

The serum fractalkine concentrations were not different between C and PF rats and were reduced in L rats compared to the other groups (Figure 1A), whereas IFN-γ and IL-2 were increased in L rats (Figure 1B,C, respectively). The IL-6 concentrations remained unchanged (Figure 1D), and TNF-α levels were decreased in PF and L rats, being lower in the L group (Figure 1E). The IL-4 concentrations did not change in the PF group and were augmented in L rats (Figure 1F), and IL-10 presented no differences among the experimental groups (Figure 1G). The serum levels of adiponectin and irisin were only increased in L rats compared to the other groups (Figure 1H,I, respectively).

### 3.3. Leptin Reduces the Activation of Inflammatory Targets in Gastrocnemius

**Figure 2:**
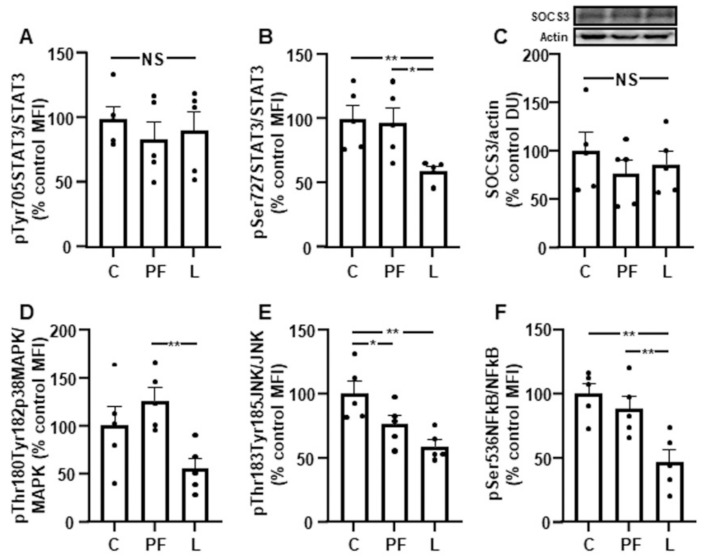
Effect of chronic central leptin infusion on activation of leptin and inflammatory targets. Relative protein levels of (**A**) signal transducer and activator of transcription 3 (STAT3) phosphorylated (p) on Tyr705 (pTyr705STAT3), (**B**) phosphorylated STAT3 on Ser727 (pSer727STAT3), (**C**) suppressor of cytokine signaling (SOCS)3, (**D**) phosphorylated p38 mitogen-activated protein kinase (p38MAPK) on Thr180 and Tyr182 (pThr180Tyr182p38MAPK), (**E**) c-Jun N-terminal kinase (JNK) phosphorylated on Thr183 and Tyr185 (pThr183Tyr185JNK) and (**F**) nuclear factor kappa B (NFkB) phosphorylated on Ser536 (pSer536NFkB) in the gastrocnemius of control rats (C), pair-fed rats (PF) and rats receiving chronic intracerebroventricular leptin infusion (L). Data are presented as means ± SEM. DU, densitometry units; MFI, median fluorescent intensity; NS, non-significant. * *p* < 0.05, ** *p* < 0.01; one-way ANOVA followed by Bonferroni’s test. Dots represent the individual data.

Pair feeding did not modify the phosphorylation of inflammatory targets, with the exception of JNK. The phosphorylation of STAT3 on Tyr705 remained unaffected (Figure 2A), and the phosphorylation of STAT3 on Ser727 was diminished in L rats compared to the other groups (Figure 2B). SOCS3 protein levels presented no differences (Figure 2C), and p38MAPK phosphorylation was reduced in the L group compared to in PF rats (Figure 2D). The phosphorylation of JNK was decreased in both PF and L rats, being more marked in the L group (Figure 2E), while NFκB was decreased in the L group compared to C and PF rats (Figure 2F).

### 3.4. Leptin Infusion Decreases the Inflammatory Pattern in Gastrocnemius

**Figure 3:**
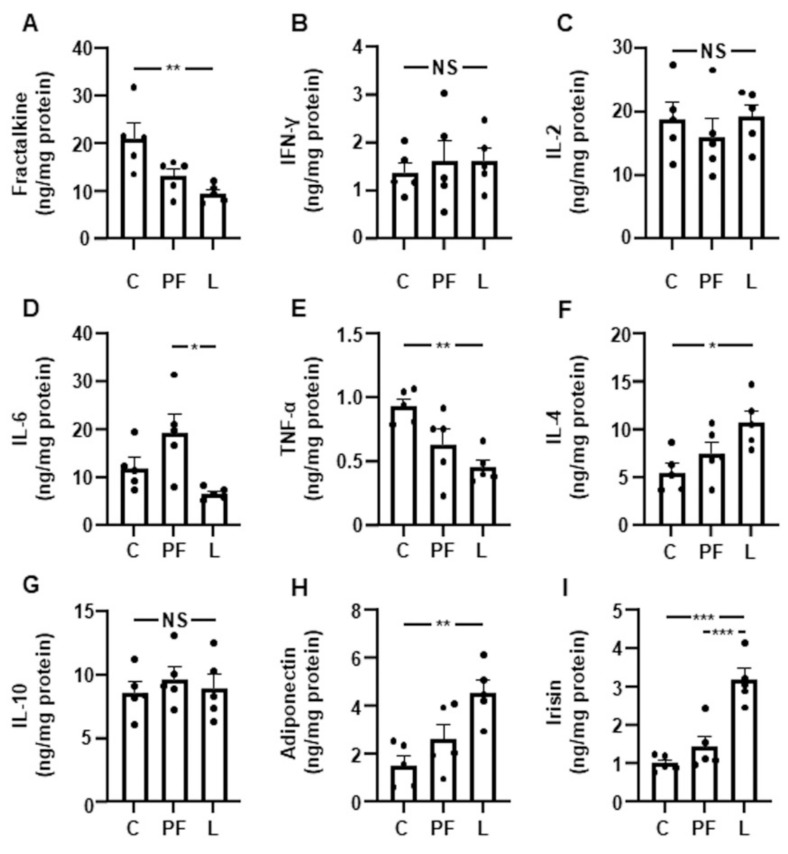
Effect of leptin on muscle cytokine levels. Fractalkine (**A**), interferon (IFN)γ (**B**), interleukin (IL)2 (**C**), IL-6 (**D**), tumor necrosis factor (TNF)α (**E**), IL-4 (**F**), IL-10 (**G**), adiponectin (**H**) and irisin (**I**) content in the gastrocnemius of control rats (C), pair-fed rats (PF) and rats receiving chronic intracerebroventricular leptin infusion (L). Data are presented as means ± SEMNS, non-significant. * *p* < 0.05, ** *p* < 0.01, *** *p* < 0.001; one-way ANOVA followed by Bonferroni’s test. Dots represent the individual data.

A reduction in the fractalkine levels was detected in the gastrocnemius of L rats (Figure 3A), whereas the IFNγ and IL-2 concentrations remained unchanged (Figure 3B,C, respectively). The levels of IL-6 were increased in PF rats with respect to the L group (Figure 3D), and TNF-α was only reduced in L rats (Figure 3E). The IL-4 levels were unchanged in PF rats and were augmented in the L group (Figure 3F), and no differences in the IL-10 concentrations were found (Figure 3G). Pair feeding did not change the adiponectin and irisin levels in gastrocnemius; however, these factors were augmented in the L group (Figure 3H,I, respectively).

### 3.5. Chronic Leptin Infusion Increases IRS1/PI3K Signaling

**Figure 4:**
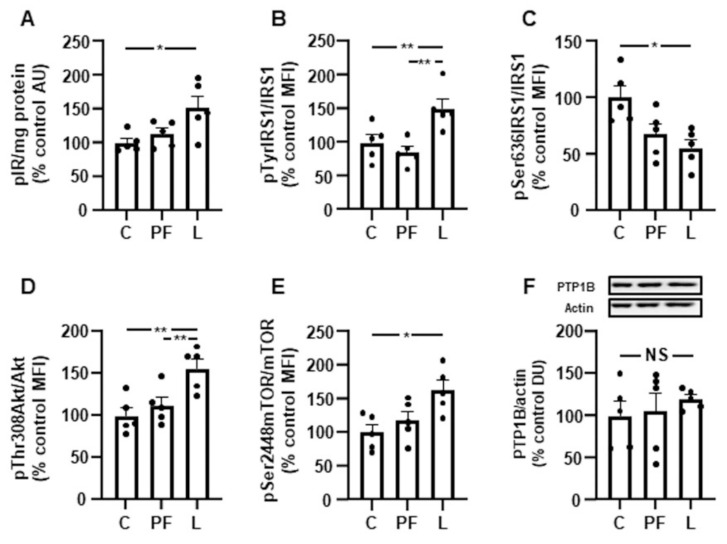
Leptin infusion increases Akt signaling. Relative protein levels of (**A**) phosphorylated (p) insulin receptor (pIR), (**B**) insulin receptor substrate 1 (IRS1) phosphorylated on Tyr residues (pTyrIRS1), (**C**) insulin receptor substrate 1 (IRS1) phosphorylated on Ser636 (pSer636IRS1), (**D**) Akt phosphorylated on Thr308 (pThr308Akt), (**E**) mammalian target of rapamycin (mTOR) phosphorylated on Ser2448 (pSer2448mTOR) and (**F**) protein tyrosine phosphatase 1B (PTP1B) in the gastrocnemius of control rats (C), pair-fed rats (PF) and rats receiving chronic intracerebroventricular leptin infusion (L). Data are presented as means ± SEM. AU, absorbance units; DU, densitometry units; MFI, median fluorescent intensity; NS, non-significant. * *p* < 0.05, ** *p* < 0.01; one-way ANOVA followed by Bonferroni’s test. Dots represent the individual data.

Insulin-related signaling remained unchanged in the PF group. The phosphorylation of the insulin receptor was increased in the L group (Figure 4A), and IRS1 phosphorylation on Tyr residues was more augmented in the L group compared to C and PF rats (Figure 4B). IRS1 phosphorylation om the Ser636 residue was reduced in L rats (Figure 4C). Akt activation, measured as phosphorylation on Thr308, was augmented in the L group (Figure 4D), and mTOR phosphorylation was increased in L rats (Figure 4E). The relative PTP1B protein levels did not present significant differences (Figure 4F).

### 3.6. Leptin Changes Markers of Lipid Metabolism and NEFA Levels in Gastrocnemius

**Figure 5:**
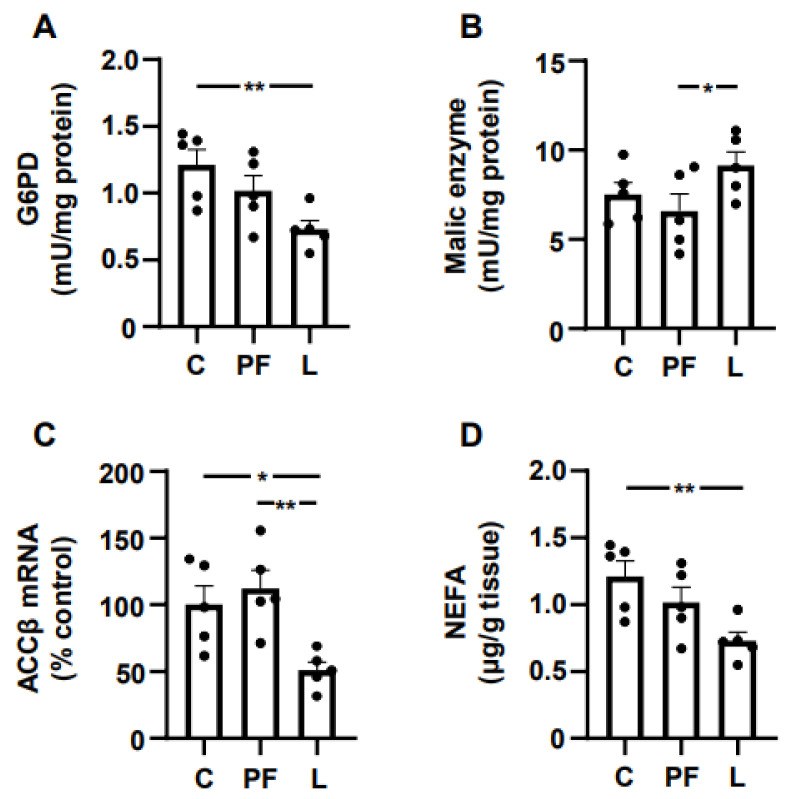
Changes in lipid metabolism markers after leptin infusion. Activity of (**A**) glucose 6-phosphate dehydrogenase (G6PD) and (**B**) malic enzyme, (**C**) acetyl-CoA carboxylase (ACC)β mRNA levels and (**D**) non-esterified fatty acid (NEFA) content in the gastrocnemius of control rats (C), pair-fed rats (PF) and rats receiving chronic intracerebroventricular leptin infusion (L). Data are presented as means ± SEM. * *p* < 0.05, ** *p* < 0.01; one-way ANOVA followed by Bonferroni’s test. Dots represent the individual data.

The activity of G6PD did not change in PF rats and was decreased in L rats (Figure 5A). The malic enzyme activity increased in L rats compared to the PF group (Figure 5B). The relative mRNA content of ACCβ was only reduced in the L group with respect to C and PF rats (Figure 5C). The tissue levels of NEFA in PF rats were not different from controls and were diminished in L rats (Figure 5D). 

### 3.7. Inflammation Is Inversely Related to Irisin Levels and Akt Activation

**Figure 6:**
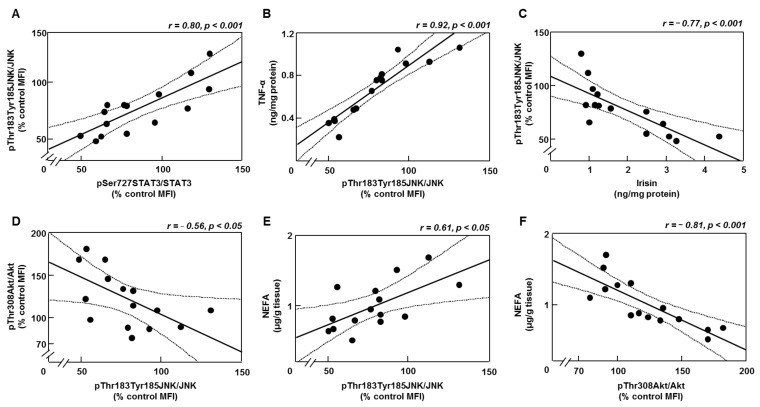
Correlations between leptin-, inflammatory- and insulin-related targets in gastrocnemius. (**A**) Linear correlations between relative protein levels of c-Jun N-terminal kinase (JNK) phosphorylated on Thr183 and Tyr185 (pThr183Tyr185JNK) and signal transducer and activator of transcription 3 (STAT3) phosphorylated (p) on Ser727 (pSer727STAT3), (**B**) tumor necrosis factor (TNF)α and pThr183Tyr185JNK, (**C**) pThr183Tyr185JNK and irisin, (**D**) Akt phosphorylated on Thr308 (pThr308Akt) and pThr183Tyr185JNK, (**E**) non-esterified fatty acid (NEFA) content and pThr183Tyr185JNK and (**F**) NEFA and pThr308Akt. MFI, median fluorescent intensity. The 95% confidence interval is indicated by hatched curves. Correlation coefficients (r) and *p* values are represented for each analysis. Dots represent the individual data.

Leptin is implicated in the regulation of several inflammatory markers that can reduce insulin signaling and insulin-sensitizing molecules [27,28]. Linear regression analyses showed positive correlations of pJNK with pSTAT3, TNF-α and NEFA levels in the gastrocnemius (Figure 6A,B,E, respectively) and inverse correlations with irisin and pAkt in the same localization (Figure 6C,D, respectively). Muscle NEFA concentrations were inversely related to pAkt (Figure 6F).

## 4. Discussion

Leptin reduces glycemia and has anti-lipogenic effects that are unrelated to leptin’s regulation of body weight. This adipokine acts directly in muscles via both long and short isoforms of the leptin receptor, raising fatty acid uptake and oxidation and lessening triglyceride synthesis [29]. Our data provide in vivo evidence that increased central leptin levels can augment insulin signaling in gastrocnemius and that this seems to be associated with an anti-inflammatory profile and a decline in NEFA levels. Furthermore, most of the effects of leptin differed from those in pair-fed rats and thus were most likely not due to the decrease in food intake or weight loss. Lastly, our results suggest that gastrocnemius may impact the peripheral levels of several cytokines, particularly in insulin-sensitizing molecules.

Most of the observed effects did not appear to be due to a decrease in weight or food intake but to a direct effect of leptin. However, we detected a decrease in the circulating levels of TNF-α and also in muscle JNK activation in the pair-fed group. The decrease in the blood concentration of TNF-α in pair-fed rats could have been due to a lower expression of this cytokine in other localizations, such as subcutaneous adipose tissue [30], while the increase in insulin sensitivity reduces the phosphorylation of JNK [31] and, in this way, calorie restriction prevents the development of insulin resistance in rat skeletal muscle [32]. We found no differences in the serum IL-6 concentrations among the experimental groups, in spite of the augmentation in IL-6 content in the gastrocnemius of pair-fed rats compared to the leptin group. This could have been due to a direct effect of leptin in other localizations, as chronic leptin infusion reduces IL-6 content in other tissues [24], which could counteract the possible contribution of muscle to the circulating levels of this interleukin.

One of the most remarkable findings was the decrease in some inflammatory cytokines in the muscle of leptin-infused rats, whereas this pattern differed in the circulating levels exhibited by this experimental group. Thus, leptin produces tissue-specific effects on the expression of cytokines. In general, it induces an inflammatory state in adipose tissue which affects the systemic circulation [30], while in other organs, it generates an anti-inflammatory profile that could offset the actions exerted by fat depots [33]. Although leptin may be considered as a proinflammatory cytokine, the role of leptin has been largely explored in pathological conditions, such as metabolic syndrome and obesity, where there is an increase in proinflammatory interleukins related to leptin resistance [34]. Local monocytes and macrophages in muscle are inactive, but when damage happens, activated macrophages attract other cells that may express proinflammatory cytokines, whereas M2 macrophages are involved in the synthesis of anti-inflammatory interleukins that participate in muscle recovery [35]. Leptin also exerts beneficial effects against lipid accumulation in several organs such as muscle and liver, where there are resident macrophages [36]. A reduction in STAT3 phosphorylation could inhibit inflammatory pathway cascades; indeed, STAT3 phosphorylated on Ser727 activates p38MAPK and JNK, required for the synthesis of proinflammatory cytokines [37], whereas a reduction in the phosphorylation of these targets after leptin infusion could be related to the decline in muscle inflammatory cytokines observed here. Likewise, constitutive STAT3 phosphorylation is linked to the development of insulin resistance in skeletal muscle [38]. An increase in adiponectin and irisin in the gastrocnemius of leptin-treated rats could reduce the activation of inflammatory targets and generate an anti-inflammatory profile, as previously reported [39,40].

Lipotoxicity is usually linked to insulin resistance in skeletal muscle. Muscle lipid abnormalities are implicated in the activation of protein kinase C and JNK activation, which impair insulin-stimulated signaling. In addition, acting through NFκB, it can promote inflammatory pathways with a simultaneous synthesis of proinflammatory cytokines [41]. Insulin resistance in the muscle of non-obese normoglycemic subjects is related to JNK activation connected to increased intramyocellular lipids and IRS-1 serine phosphorylation [42]. This phosphorylation interferes with insulin signaling by favoring the degradation of this molecule and decreasing signal transduction. We found a decline in NEFA content in serum and muscle after leptin treatment and a rise in the activation of insulin signaling. Our data showed an augmentation in insulin receptor phosphorylation, with a concomitant reduction in the phosphorylation of IRS1 on serine residues and an increase in tyrosine after leptin infusion. The addition of leptin to myogenic cells stimulates the insulin response by increasing insulin receptors and IRS-1 phosphorylation [43]. The activation of this target allows the activation of Akt though phosphorylation on Thr308 residues, required for the activation loop via PI-dependent kinase-1 and the phosphorylation of downstream signaling targets [3].

The reduction in NEFA levels in the gastrocnemius after leptin infusion could also have been due to the reduction in some markers of lipid anabolism, such as G6PD and ACCβ; in fact, central leptin decreases triacylglyceride levels and lowers lipogenesis [44]. The increase in malic enzyme activity after leptin infusion could indicate a leptin effect independent of a reduction in food intake and related to higher insulin sensitivity that modulates this enzyme [45].

The Akt pathway also modulates cytokine synthesis. We found an increase in mTOR phosphorylation in leptin-treated rats, and mTOR complex 1 was shown to limit the activation of immune cells by raising anti-inflammatory cytokines and reducing those proinflammatory cytokines [46]. The reduction in inflammatory cytokines could be associated with a probable decline in FOXO1 content. After mTOR activation, FOXO1 phosphorylation and translocation to the cytosol is accelerated where it is degraded. This fact reduces the synthesis of proinflammatory factors mediated by FOXO1 [47], and moreover, the upregulation of insulin signaling increases IL-4 production, favoring the degradation of FOXO1 [48].

Several modifications in circulating cytokines and myokines, and particularly in the production of the fibronectin type III domain-containing protein 5 (FNDC5), irisin, could indicate that skeletal muscle contributes to these changes. Although adipose tissue and the liver are two of the organs that largely control the serum levels of cytokines, skeletal muscle influences the peripheral levels of these molecules. Myokines have been studied to a lesser degree than adipokines, but skeletal muscle is an active player, secreting different interleukins and myokines, improving glucose homeostasis and reducing lipid accumulation in different tissues [49]. Indeed, in obese patients, FNDC5 expression in skeletal muscle and peripheral irisin concentrations are reduced and associated with changes in insulin sensitivity [50].

A couple of aspects should be considered when evaluating these results. We determined the NEFA levels in skeletal muscle, but not the content of several products, such as ceramide, which are involved in the generation of insulin resistance in the gastrocnemius [51]. Another caveat is that we only analyzed the content of cytokines and not their expression in muscle in order to more precisely measure how this muscle might contribute to peripheral cytokine patterns.

## 5. Conclusions

Our findings sustain the concept that the reduction in muscle NEFA content after chronic leptin infusion is related to an anti-inflammatory profile in skeletal muscle which could modify intracellular insulin signaling. These modifications seem to be due to a direct leptin effect independent of a reduction in food intake and weight loss, suggesting that leptin may integrate both pathways.

## 6. Future Prospects

Leptin is emerging as an important player in diverse body functions, and in situations without leptin resistance, this adipokine may regulate inflammatory signaling and related cytokine expression in different organs. In fact, although leptin can increase some circulating inflammatory markers, in particular, those derived from fat depots, it also exerts positive effects on insulin sensitivity in numerous tissues. This key role of leptin on the increase in insulin actions are often related to an improvement in the lipid content and the inflammatory environment in these organs, which could contribute to the generation of a systemic anti-inflammatory profile. Thus, a logical line of investigation in leptin studies is to explore the molecular mechanisms involved in the improvement of insulin signaling in different tissues, such as the gastrocnemius and its association with changes in the expression of cytokines. Future studies will verify in depth of the connection between modifications in lipid metabolism and inflammation targets together with changes in the PI3K/Akt signaling pathway.

## Data Availability

Data is contained within the article.
